# Inside Enemy Lines: Adhesion, Invasion, and Intracellular Persistence of *Acinetobacter baumannii* in the Respiratory Epithelium

**DOI:** 10.3390/pathogens15010102

**Published:** 2026-01-19

**Authors:** Dolores Limongi, Daniela Scribano, Anna Teresa Palamara, Cecilia Ambrosi

**Affiliations:** 1 Department of Promotion of Human Sciences and Quality of Life, San Raffaele Open University, Via di Val Cannuta, 247, 00166 Rome, Italy; dolores.limongi@uniroma5.it; 2 Laboratory of Microbiology, Istituto di Ricovero e Cura a Carattere Scientifico (IRCCS) San Raffaele Roma, 00166 Rome, Italy; 3 Department of Public Health and Infectious Diseases, Sapienza University of Rome, 00185 Rome, Italy; daniela.scribano@uniroma1.it; 4 Department of Infectious Diseases, Istituto Superiore di Sanità, 00185 Rome, Italy; annateresa.palamara@iss.it; 5 Department of Public Health and Infectious Diseases, Sapienza University of Rome, Laboratory Affiliated to Institute Pasteur Italia-Cenci Bolognetti Foundation, 00185 Rome, Italy

**Keywords:** *Acinetobacter baumannii*, adhesion, invasion, intracellular persistence, human respiratory epithelium, host adaptation

## Abstract

*Acinetobacter baumannii* is a critical pathogen and a leading cause of hospital-acquired pneumonia, especially in immunocompromised patients. Although most research has focused on antimicrobial resistance, growing evidence shows that *A. baumannii* can efficiently adhere to, invade, and persist within human airway epithelial cells. Thus, the aim of this review is to summarize current knowledge on the mechanisms used by *A. baumannii* to establish infection, highlighting the bacterial traits responsible for attachment to airway epithelia, entry into host cells, manipulation of intracellular trafficking pathways to avoid degradation, metabolic adaptation to the host environment, and interference with immune defenses. The findings reported herein come from host–pathogen studies performed using epithelial cell lines, *Galleria mellonella*, and murine models, and from human primary airway cells. Despite the prominent role of the outer membrane protein OmpA, it is clear that *A. baumannii* pathogenicity relies on multiple, often redundant, virulence strategies to secure its intracellular niche and resist host pressures. Remarkably, strain heterogeneity in virulence traits between lab-domesticated and clinical isolates supports differential intracellular behavior and pathogenic potential. A deeper understanding of *A. baumannii* infection mechanisms is essential to design anti-virulence strategies that disarm this life-threatening bacterium, reduce selective pressure, limit resistance, and guide next-generation therapeutic interventions.

## 1. Introduction

### A. baumannii: Breathing Trouble into the Airways

*A. baumannii* is a Gram-negative, strictly aerobic, non-fermentative, oxidase-negative, and catalase-positive coccobacillus [1,2,3]. Since the 1960s, it has shifted from a bacterium of questionable pathogenicity to a critical World Health Organization-listed priority pathogen for healthcare settings [4]. The main mechanism that turned *A. baumannii* into a global menace lies in its extraordinary ability to acquire and accumulate multiple resistance mechanisms via horizontal gene transfer, including integration of mobile elements, and upregulation of several antibiotic resistance determinants [5,6,7]. These mechanisms directly and indirectly underlie the emergence of multidrug-resistant (MDR), extensively drug-resistant (XDR), and pandrug-resistant (PDR) clinical strains, resistant to most if not all commercially available antibiotics, including carbapenems, cefiderocol, tigecycline and colistin [5,8,9,10]. Moreover, these strains exhibit resistance to disinfectants, and high tolerance to desiccation, oxidative stress, and host immune responses [8,11]. Together with their ability to form biofilms, these features enable *A. baumannii* to stably and successfully fit the nosocomial environment, becoming endemic in healthcare facilities and being responsible for a spectrum of infections, such as pneumonia, bacteremia, urinary tract, wound, and burn infections, especially in immunocompromised patients [5,8,11]. Consequently, it seems that *A. baumannii* clinical strains co-evolved with infection-control measures, exploiting invasive medical devices, and spreading efficiently among susceptible hosts, thereby shifting from a low-ranked opportunistic pathogen into a highly successful human pathogen. Worldwide, hospital-acquired pneumonia caused by *A. baumannii* is most frequently associated with endotracheal intubation and mechanical ventilation, with prevalence rates ranging from 65% in Eastern Asia to 100% in Central and Latin America, and an estimated overall mortality rate of 42.6% [8]. Beyond the hospital setting, *A. baumannii* also causes severe seasonal community-acquired pneumonia, which, although less studied, is characterized by alarming mortality rates often exceeding 60% [8]. Risk factors such as prolonged hospital stay and underlying comorbidities further exacerbate morbidity and mortality [8]. The combination of the alarming shortage of new antibiotic molecules in the pipeline and the rapid emergence of resistance to existing treatments highlights the urgent need for alternative therapeutic approaches. A promising paradigm shift is the development of anti-virulence drugs, aiming not to kill the bacterium directly but to disarm it, attenuating its pathogenic mechanisms, while reducing selective pressure for resistance. In this context, understanding the molecular basis of *A. baumannii* interaction with the airway epithelium is crucial for discovering new therapeutic targets and developing novel anti-virulence approaches against this pathogen. Therefore, this review aims to provide a comprehensive overview of the current knowledge on the mechanisms and virulence factors that mediate *A. baumannii* adhesion, invasion, and persistence within respiratory epithelial cells, highlighting their potential as novel therapeutic targets for innovative therapeutic approaches.

## 2. Sticking Around: *A. baumannii* Grips the Epithelium

### 2.1. Fingertips on the Surface: Pili and Fimbriae-Mediated Adhesion

**Figure 1:**
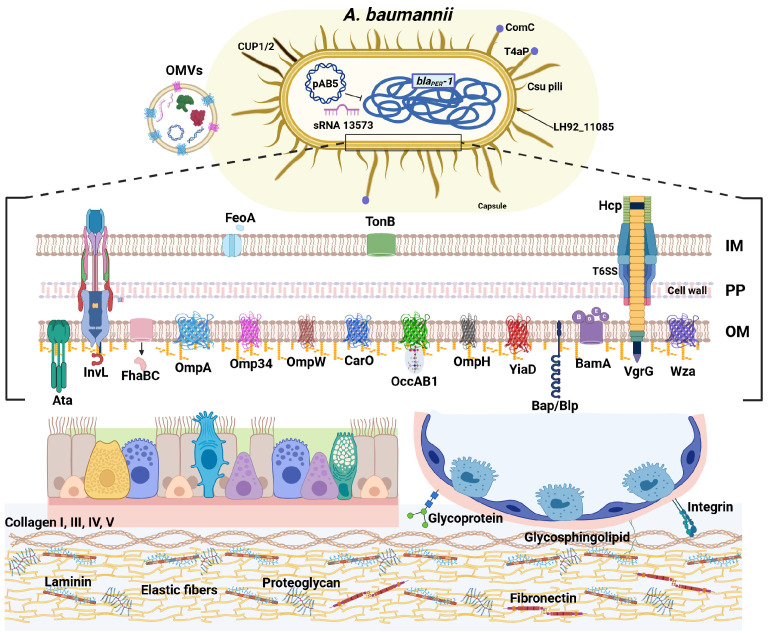
***A. baumannii* adhesion to epithelial cells.** The figure schematically illustrates the highly redundant repertoire of adhesins and surface components used by *A. baumannii* to establish a stable and multifocal anchoring with the host respiratory epithelium and ECM. Bacterial factors: Csu pili, Csu type LH92_11085, CUP1 and CUP2 pili (pAB5-regulated), and T4aP pili with the tip adhesin ComC; OMPs and autotransporters include OmpA, Omp34, OmpW, CarO, OccAB1, OmpH, and YiaD, the autotransporter Ata, the biofilm protein Bap, the adhesin InvL, and the two-partner secretion system proteins AbFhaB/FhaC (FhaBC). The presence of the *bla_PER-1_* gene positively correlates with bacterial adhesion abilities. Secreted and regulatory components comprise the non-coding sRNA 13573, the iron transport component FeoA, T6SS components Hcp and VgrG, the capsule export protein Wza, the regulatory plasmid pAB5, and OMVs enriched in adhesins, like Omp34 and TonB-dependent copper receptor. Host receptors and ECM targets: fibronectin, collagens I, III, IV, and V, laminin, integrins (e.g., α5β1), glycosphingolipid receptors and glycoproteins. OM, outer membrane; PP, periplasmic space; IM, inner membrane. Results herein summarized relate to studies performed in bronchial and lung cell infection models. Figure, not drawn to scale, was created with BioRender.com.

Successful colonization of the respiratory epithelium starts with the ability of *A. baumannii* to adhere to host cells, a critical first step that determines the course of infection and facilitates subsequent invasion and persistence. Early studies suggested that the ability of *A. baumannii* to colonize and persist in the host relies heavily on its firm and stable adhesion to human airway epithelial cells (Figure 1) [12,13]. Accordingly, at that time, in vitro studies reported that *A. baumannii* clinical isolates displayed two different patterns of adherence [12]. Since most studies focused mainly on antibiotic-resistance genes, it was also demonstrated that bacterial adhesion abilities correlated positively with the presence of the *bla_PER-1_* gene in MDR clinical isolates of *A. baumannii*, highlighting a possible link between resistance determinants and virulence traits [13]. Intriguingly, a specific small RNA non-coding transcript of the lab-domesticated strain ATCC 17978, sRNA 13573, probably under the control of the quorum sensing system, was upregulated during adhesion to human lung epithelial cells; although not clarified yet, it has been suggested that it causes the inhibition of *csuE* expression, leading to the abolition of pili production and biofilm formation, which enhances epithelial adhesion [14]. In line with the observation that biofilm and host cell adhesion are often intertwined features, the well-known biofilm-associated protein (Bap) was found to be involved in initial adhesion to primary human bronchial, although it did not contribute to internalization [15]. Similarly, in the hyper-biofilm producing strain MAR002, overexpression of the gene *LH92_11085* encoding a Csu type fimbrial protein homologous to the *Escherichia coli* type I pilus subunit FimA, promoted adhesion to human alveolar epithelial cells by forming long pili [16]. Notably, this gene is highly conserved across *A. baumannii* genomes available at the National Center for Biotechnology Information (NCBI). Beyond their role in robust biofilm formation and pellicle development, the *A. baumannii* Csu pilus system represents major virulence factors in host interaction [17]. Under the control of c-di-GMP signaling, bacterial adherence to epithelial cells and systemic colonization in vivo depend on the expression of Csu pili, which are inversely influenced by the phosphodiesterase PdeB in strains ATCC 17978 and AB5075 [17]. Notably, PdeB also promotes the expression of shorter pili, type IVa pili (T4aP), which are essential for both twitching motility and natural competence [17]. Consistent with this regulatory interplay, T4aP have also been implicated in mediating adhesion to host epithelial cells [18,19]. Variations in the domain architecture of the tip adhesin ComC, such as the acquisition of a von Willebrand A domain, modulate strain-specific host recognition and binding efficiencies [18,19].

### 2.2. The Molecular Velcro: Host–Pathogen Protein Interactions

Another key player in the interaction with host cells is the trimeric autotransporter adhesin Ata, which binds to heavily glycosylated extracellular matrix (ECM) components, including galactose, N-acetylglucosamine, and galactose (β1−3/4) N-acetylglucosamine [20,21]. Thus, the Ata lectin activity mediates high-affinity binding to collagen (types I, III, IV, and V), laminin, and fibronectin [21]. In uropathogenic *A. baumannii*, the unique member of the intimin–invasin family of proteins InvL has been identified [22]. InvL mediates direct binding to multiple ECM components; its lectin-like domain and immunoglobulin-like motifs allow binding to α5β1 integrin, collagen type V, respectively, as well as fibronectin, thereby granting multiple and intimate interactions with host epithelial cells [22]. This highly insoluble adhesin is predicted to be a lipoprotein that is secreted by the type II secretion system (T2SS), raising the hypothesis that it could be a component of the bacterial outer membrane vesicles (OMVs) [22]. Despite its binding activities, to date, no direct evidence supports a role for InvL in epithelial cell invasion. Notably, its presence appears to be highly strain-dependent, being detected in certain *A. baumannii* uropathogens, but not being widespread across clinical isolates [23]. Interestingly, in the XDR uropathogenic UPAB1 strain, the large conjugative plasmid pAB5 was shown to modulate the expression of multiple chromosomally encoded virulence factors involved in host cell adhesion (e.g., CUP1 and CUP2 pili, OmpW), as well as metabolism, biofilm formation, and protein secretion systems [24]. Furthermore, the filamentous hemagglutinin proteins of the two-partner secretion system AbFhaB/FhaC also contribute to fibronectin binding in the clinical isolate AbH12O-A2, further emphasizing the importance of the adhesion to the ECM in *A. baumannii* pathogenesis [25]. Beyond specific protein-glycan interactions, additional *A. baumannii* adherence mechanisms extend to its recognition of glycosphingolipid receptors on host cell surfaces, particularly those containing GlcNAc, thereby broadening the possibilities of interaction with host epithelia [26]. Among the numerous outer membrane proteins (OMPs) described in *A. baumannii*, the outer membrane protein A (OmpA) stands out as the most extensively studied and pivotal, given its multifaceted contributions to both bacterial physiology and pathogenesis; this pleiotropic protein is involved in maintaining cell envelope integrity, antibiotic-and serum resistance, biofilm formation as well as in pathogenesis and immune evasion [15,27,28,29,30,31,32]. Several lines of evidence highlighted the crucial role of OmpA, also known as Omp38, in the interaction with the human host epithelium [13,15,31,33,34,35,36]. OmpA has been shown to interact directly with fibronectin, which facilitates bacterial adhesion to epithelial cells [34]. Recently, the most plausible amino acids mediating the interaction of the OmpA-fibronectin complex were provided; through this interaction, *A. baumannii* not only enhances its ability to anchor to host surfaces but also gains a protective advantage, as fibronectin binding can shield the bacterium from immune recognition, thereby promoting persistence and favoring the dissemination of infection [37]. Besides OmpA, other porins can mediate host cell adhesion, such as Omp34 and the TonB-dependent copper receptor that are able to bind to the fibronectin in addition to being essential for bacterial physiology and antibiotic-resistance [34,38,39,40]. Omp34 was also found to be involved in the alteration of the cell membrane, apoptosis, vacuolization, and cell damage, granting bacterial intracellular proliferation in human cervical epithelial cells [39,41]. In addition to biofilm formation, the OmpW protein contributes both to epithelial adhesion and invasion and to antibiotic-resistance in *A. baumannii* persister cells in an animal model [42,43,44]. Historically linked to carbapenem-resistance, CarO is the second most abundant porin of *A. baumannii* [45]. Due to significant mutations of the *carO* gene, four different variants have been reported among *A. baumannii* clinical isolates [46]. This protein has been implicated in multiple aspects of *A. baumannii* pathogenesis. It is involved in adhesion to and invasion of epithelial cells [35,47,48], and it also supports the bacterial physiology under iron-limiting conditions, during biofilm formation, and as a channel for basic amino acids, suggesting an active role also during the bacterial intracellular lifestyle [48]. Among the five orthologs of the outer membrane carboxylate channels (Occ) involved in the transport of small molecules [49], OccAB1 (also known as OprD) is the best characterized in the context of host interaction, as the full length of this protein has been associated with adhesion to and invasion of human lung epithelial cells and with the hypervirulence of carbapenem-resistant isolates [50,51,52] (see below). Recently, the YiaD porin associated with carbapenem-resistance (mainly meropenem) was shown to be involved in adhesion to pulmonary epithelial cells [35,53]. Additional and hypothetical adhesins were suggested to be involved in adhesion to host cells, such as the β-barrel assembly machinery A (BamA, see below), OmpH, fimbrial protein F (FimF), pilus assembly protein, the TonB-dependent copper receptor (either membrane-bound or associated with OMVs), and the Bap-A-prefix domain-containing protein adhesin [40,54,55,56]. In addition, FeoA, the component of the ferrous iron transport system (*feoABC*), was shown to be upregulated during infection in vivo both in *A. baumannii* strains ATCC 17978 and AbH12O-A2 [57]. Deletion of the *feoA* gene markedly reduces bacterial adhesion to and invasion of the A549 human lung adenocarcinoma cell line (see below for invasion) [57]. In addition, non-targeted metabolomic analyses performed on three components of the co-culture system, the supernatant, the host cells, and *A. baumannii* ATCC 17978 and its *hcp* or *vgrG* type VI secretion system (T6SS) isogenic mutants, revealed specific bacterial metabolic responses and a significant reduction in host cell adhesion, thereby highlighting the functional link between the T6SS, host interaction, and bacterial metabolic adaptation [58].

### 2.3. The Sticky Coat: Capsule-Mediated Adhesion

In *A. baumannii* ATCC 17978 and the highly virulent clinical isolate SKLX024256, the capsule export protein Wza, belonging to the Wzy-dependent capsular polysaccharide synthesis pathway, has been shown to play a pivotal role in *A. baumannii* adhesion to A549 lung epithelial cells, as well as in serum resistance and in vivo virulence in both *G. mellonella* and murine models [59]. Interestingly, *wza* knockout strains revealed that the expression of other capsular genes (e.g., *wzb*, *wzc*, and *wzi*) is also under the control of Wza [59]. Thus, these data indicate that Wza not only mediates capsule assembly and export, but also indirectly modulates epithelial adhesion by coordinating the expression of capsular biosynthesis genes [59]. Recently, Debruyne et al. analyzed the growth-phase dependency of the invasive ABC141 strain on the adhesion, invasion and intracellular replication within lung epithelial cells; results showed that the twin-arginine translocation (Tat) export system during the exponential growth is required for adhesion to lung epithelial cells, although it does not appear to be involved in the invasion process [60].

Bacterial adhesion is known to be a multifactorial process, mediated by a dynamic interplay between bacterial and host factors. On the bacterial side, diverse and redundant structures often act in synergy, ensuring robust colonization under different host conditions. In *A. baumannii*, the relative contribution of each adhesin varies among clinical isolates, reflecting its genomic plasticity. This feature explains the heterogeneity of adhesion phenotypes observed in vitro and in vivo, yet the precise molecular interplay and regulation of these adhesion mechanisms remain incompletely understood. On the host side, adhesion of *A. baumannii* AB5075 on advanced airway epithelial models triggers early tissue alterations, detectable as soon as 4 h post-infection, progressing to goblet cell hypertrophy, reduced mucin secretion, and epithelial disruption [61]. Transcriptomic data showed that *A. baumannii* triggers a strong IL-8/CCL20-driven pro-inflammatory response and type 2 cytokine activation (IL-4, IL-13); genes involved in cytoskeletal organization, adhesion, and ECM remodeling were significantly affected, consistent with a bacterial strategy to promote tissue dissemination [61]. Collectively, these findings suggest that *A. baumannii* exploits both structural components (such as OMPs) and secreted effectors (OMVs) to reprogram host signaling and metabolic pathways, thereby promoting epithelial injury and facilitating bacterial persistence.

## 3. Breaking in: *A. baumannii* Intracellular Entry

### 3.1. Zipping in: The Mechanism of Bacterial Entry

**Figure 2:**
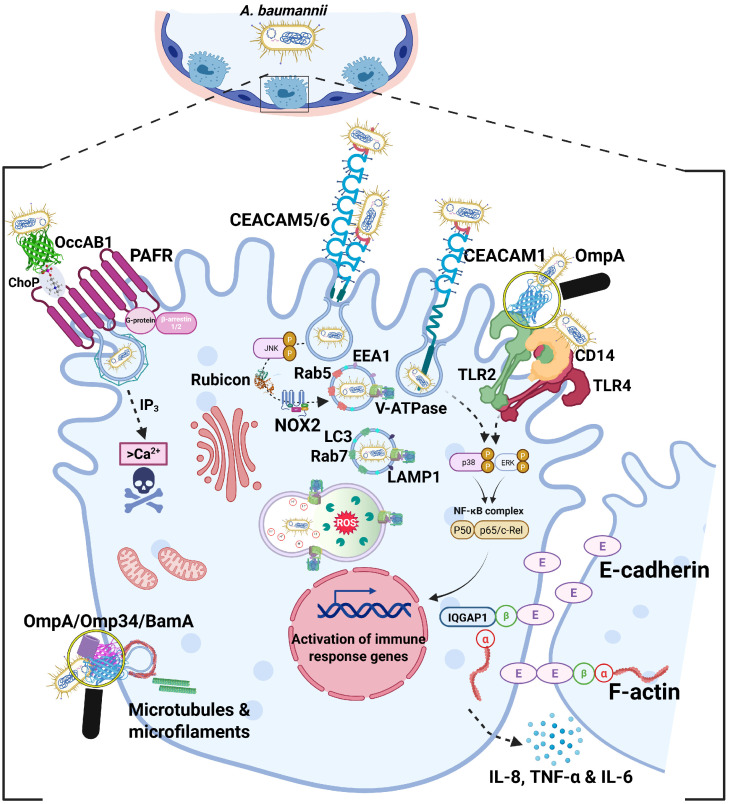
**Key steps of *A. baumannii* invasion of respiratory epithelial cells.** The figure schematically illustrates the main mechanisms underlining invasion of respiratory epithelial cells by invasive and hyperinvasive *A. baumannii* strains, highlighting the variability in intracellular outcomes. OMPs mediated invasion: OmpA, together with Omp34/BamA promotes invasion of epithelial cells through a zipper-like mechanism, that relies on microfilament- and microtubule-dependent cytoskeletal rearrangements. Host receptor-mediated invasion: Binding of OccAB1/OprD (via phosphorylcholine residues, ChoP) to PAFR induces the recruitment of cellular phospholipase C and inositol triphosphate (IP_3_) production that triggers the influx of Ca^2+^, leading to clathrin-mediated endocytosis (via β-arrestins) and cell death; CEACAM1, CEACAM5, and CEACAM6 enhance adhesion and invasion, with CEACAM5 and CEACAM6 that mediate LC3-associated phagocytosis (LAP), influencing intracellular fate. Intracellular trafficking: Internalized bacteria reside within membrane-bound vacuoles that sequentially acquire Rab5, EEA1, Rab7 and LAMP1. Then, bacterial fate is strain-dependent: vacuoles may undergo progressive acidification through V-ATPase recruitment, while intravacuolar ROS production occurs via Rubicon, which stabilizes the NOX2 complex; these vacuoles eventually mature into LC3-positive compartments that fuse with lysosomes, resulting in bacterial degradation. Alternatively, hyperinvasive strains can alkalinize the vacuole, granting intracellular replication and spread. Host immune recognition and response: TLR2 (directly interacting with OmpA) and TLR4 (using soluble CD14 as a co-receptor) activate NF-kB and MAPK pathways leading to the release of pro-inflammatory cytokines (IL-8, TNF-α, IL-6) and β-defensin 2; OmpA-TLR2 interaction also induces a signaling pathway that disrupts E-cadherin-mediated cell–cell adhesion (via IQGAP1) contributing to epithelial barrier dysfunction. Solid and dotted arrows represent direct and indirect interactions, respectively. Results herein summarized relate to studies performed in lung cell infection models. Figure, not drawn to scale, was created with BioRender.com.

After adhesion, *A. baumannii* employs several strategies to invade epithelial cells, exploiting host receptors, and signaling pathways, to establish itself within this protected niche (Figure 2). However, for many years, *A. baumannii* was regarded almost exclusively as an extracellular pathogen [62,63], even after the evidence provided by Choi and coll. in 2008; indeed, it was demonstrated that some *A. baumannii* strains were able to enter human epithelial cell lines by a zipper-like mechanism and reside in membrane-bound vacuoles, a process that exploited host microfilaments and microtubules, mediated, at least in part, by OmpA [31]. Moreover, epithelial cell lines of respiratory origin appeared to be more susceptible to *A. baumannii* entry than those derived from non-respiratory tissues, suggesting a degree of tissue specificity in the invasion process [31]. Nevertheless, discrepancies between studies using different strains and cell models fueled controversies about whether *A. baumannii* is a true facultative intracellular pathogen or whether the intracellular detection of *A. baumannii* was an uncommon or incidental finding rather than an evolved pathogenic strategy [15,31,62,63]. The first molecular evidence of a specific receptor–ligand interaction of *A. baumannii* driving adhesion to and invasion of human lung epithelial cells came from the discovery that phosphorylcholine (ChoP) residues on the porin OccAB1/OprD interacted with the platelet-activating factor receptor (PAFR), a G protein-coupled receptor on human lung epithelial cells [49,51]. This interaction not only stabilizes bacterial adhesion but also activates signaling cascades; activation of phospholipase C increases intracellular Ca^2+^ concentrations, leading to actin cytoskeletal rearrangements at the membrane. β-arrestins then link PAFR to clathrin-mediated endocytosis, thereby promoting bacterial endocytosis [51]. Despite these findings, whether *A. baumannii* can consistently replicate within host cells remains uncertain, with its intracellular presence often considered incidental rather than a defining feature of its pathogenic strategy. However, advances in comparative genomics have underscored the extraordinary plasticity of the *A. baumannii* genome, which likely explains the heterogeneous intracellular phenotypes observed among clinical isolates [64]. Differences in surface composition markedly affect the adhesion and invasion capacities of *A. baumannii* clinical strains that can be classified as non-invasive, invasive but with limited persistence inside airway epithelial host cells, and, although only a small subset, “hyperinvasive”, capable of surviving and even multiplying intracellularly [23,65,66]. In addition to PAFR, the Carcinoembryonic Antigen-Related Cell Adhesion Molecules (CEACAMs), specifically CEACAM1, CEACAM5, and CEACAM6, were shown to enhance the *A. baumannii* adhesion to and invasion of human pneumocytes, although triggering distinct host responses [67]. Interestingly, while the CEACAM1 pathway causes epithelial inflammatory signaling through fine-tuning of interleukin-8 (IL-8) secretion, CEACAM5 and CEACAM6 mediates the LC3-associated phagocytosis, a noncanonical autophagic process that may both facilitate bacterial uptake and shape the intracellular fate [67]. *A. baumannii* reference strains, including ATCC 17978, ATCC 19606, and AB5075, as well as several clinical isolates, localize within membrane-bound vacuoles [8,31,66,67,68,69]. These vacuoles are initially decorated with the early endosomal marker Rab5, which recruits Early Endosome Antigen 1 (EEA1), and subsequently with Rab7 and lysosome-associated membrane protein 1 (LAMP1). Depending on strain-specific traits, these vacuoles either mature into acidic compartments decorated with LC3, which then fuse with lysosomes and lead to bacterial killing or, alternatively, prevent lysosomal fusion by increasing vacuolar pH through ammonia production, thereby facilitating intracellular growth and bacterial escape [8,67,68,69]. Notably, both PAFR and CEACAMs are well-known entry portals for a plethora of respiratory pathogens [70]; therefore, by exploiting these same receptors, *A. baumannii* has evolved the ability to gain an intracellular niche of the airway epithelium, supporting persistence and facilitating dissemination. Given the complexity of the interaction network, the known bacterial adhesins and invasins, along with their specific host receptors and triggered mechanisms, are summarized in Table 1.

### 3.2. Knocking at the Host Cell: Porin-Mediated Signaling and Entry

*A. baumannii* cells are actively recognized by Toll-like receptor (TLR) 2 and TLR4 of lung epithelial cells, using soluble CD14 as a co-receptor [72]. The engagement and subsequent activation of these receptors induce the expression and release of interleukin (IL)-8 via nuclear factor kappa B (NF-kB) and the mitogen-activated kinases p38 and p44/42 pathways, as well as β-defensin 2, promoting neutrophil recruitment and bacterial clearance [72]. Notably, further research has shown that OmpA directly interacts with TLR2, triggering a signaling cascade that leads to increased production of pro-inflammatory cytokines, tumor necrosis factor α (TNF-α) and IL-6, via NF-kB signaling; concomitantly, OmpA enhances the association of IQGAP1 with β-catenin, which disrupts its interaction with α-catenin, and consequently weakens E-cadherin–mediated cell–cell adhesion; at the same time, OmpA reduces the IQGAP1-F-actin binding leading to cytoskeleton remodeling [73]. As a result, these alterations lead to pulmonary epithelial permeability, causing barrier dysfunction, and ultimately facilitate *A. baumannii* translocation [73]. As previously mentioned, Omp34 is an important adhesin involved in the interaction between *A. baumannii* and host epithelial surfaces [39]. More recently, Omp34 has been shown to play a direct and active role in the internalization of *A. baumannii* into human cervical carcinoma epithelial (HeLa) cells through interactions with both microfilament- and microtubule-dependent cytoskeletal components [41]. This evidence suggests that Omp34 may trigger specific signaling pathways that indirectly act on the cytoskeleton or directly induce cytoskeletal rearrangements to promote bacterial uptake [41]. Notably, the use of anti-Omp34 antibodies significantly impairs this invasive process at its early stages, highlighting the role of Omp34 as a critical virulence factor in *A. baumannii* pathogenesis [41]. Among other OMPs, BamA plays a central role in *A. baumannii* host invasion [55]. As the core component of the β-barrel assembly machinery (BAM) complex, BamA possesses a unique β-barrel structure, which can undergo conformational changes, that enable interactions with other BAM subunits and accessory lipoproteins, guiding OMP assembly and stabilizing the outer membrane [74,75,76]. However, recent evidence has highlighted a critical role for BamA in the invasion of lung epithelial cells by the *A. baumannii* clinical isolate 58ST, as proven by treatment with anti-BamA antibodies [55]. Despite the fact that specific eukaryotic interactor(s) through which BamA mediates this process remain unknown, BamA represents a very attractive target for innovative anti-bacterial therapeutic approaches [77]. Collectively, these findings indicate that *A. baumannii* should not be considered an exclusively extracellular pathogen; instead, they highlight that certain clinical strains possess a versatile and sophisticated set of molecular tools, including redundant membrane structures as well as host cell receptors, to access and exploit the epithelial intracellular niche. Accordingly, the study by Rubio et al. (2022) systematically investigated a large collection of *A. baumannii* clinical isolates and showed that, despite the fact that the majority of isolates remained extracellular, a significant subset of strains was invasive, with some being hyperinvasive, as they were able to establish a stable intracellular multiplication niche within epithelial cells [66]. Interestingly, this behavior is not limited to hospital clinical isolates, but also extends to community isolates, which exhibit comparable adhesion, invasion, and cytotoxicity abilities in epithelial cells and in vivo models to those observed in hospital isolates [78]. Indeed, the adoption of an intracellular lifestyle provides multiple selective advantages to the pathogen, offering a nutrient-rich environment sheltered from host immune defenses and antibiotic treatment, potentially allowing persistence and dissemination within the host [79].

### 3.3. Damaging from Within: OmpA- and Omp34- Cytotoxicity

**Figure 3:**
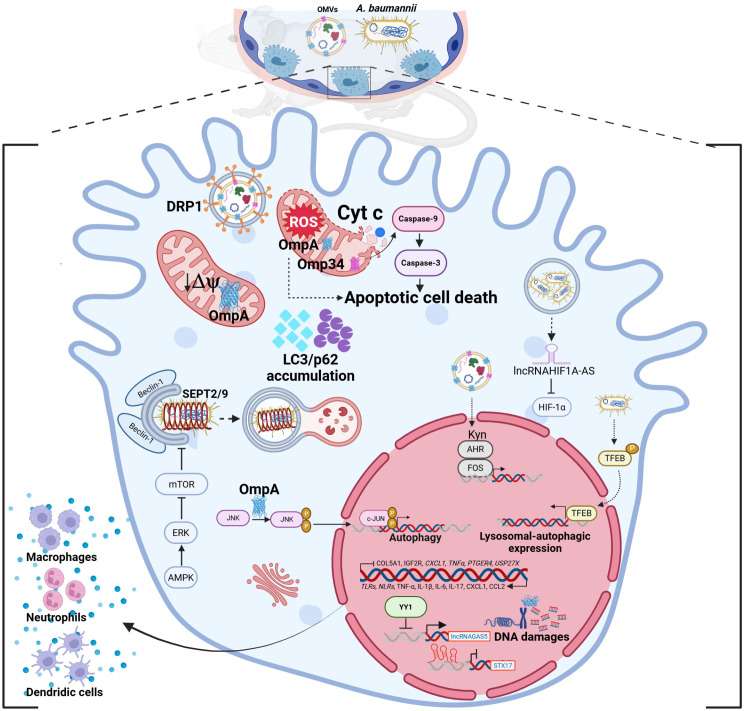
***A. baumannii* intracellular survival in respiratory epithelial cells.** The figure schematically illustrates the main intracellular strategies used by intracellular *A. baumannii* to ensure its persistence within the host cells. OMV-mediated delivery of virulence factors: following uptake of OMVs via the GTPase DRP1, OmpA is transported to mitochondria, where it dissipates membrane potential (Δψ), induces cytochrome c release, promoting ROS production, and leading to cell death. OmpA fragments can also enter the nucleus through a nuclear localization signal, where they cause DNA fragmentation and chromatin condensation, promoting apoptosis. Omp34 also induces apoptosis through activation of caspase-9 and caspase-3. Modulation of autophagy: OmpA can also activate MAPK/JNK pathways, and nuclear translocation of JNK induces autophagy via c-Jun activation; Omp34 induces accumulation of LC3-II and p62, which impairs the autophagic flux, leading to cytoplasmic vacuolization, increased cellular stress, and eventually to cell death. Bacterial factors influencing autophagy and intracellular clearance: activation of TFEB alters lysosomal biogenesis and autophagy, promoting bacterial persistence. Suppression of the normal regulation of STX17- YY1 and TFEB by lncRNA GAS5 leads to defective autophagy and inflammation. Induction of a complete, ubiquitin-dependent autophagic response by the Beclin-1/mTOR/ERK AMPK axis, involving septins SEPT2 and SEPT9. Increased host cell death is mediated by OMV components through AHR-FOS-mediated cytotoxicity. Intracellular lifestyle of hyperinvasive strains: replication within mildly acidic, LAMP1-positive single-membrane vacuoles occurs via T1SS, while T2SS is used for host cell invasion (represented). Host transcriptional reprogramming: infected cells display a hypoxia-like response characterized by upregulation of the lncRNA HIF1A-AS, which suppresses HIF1α activation. Downregulation of *COL5A1* and *IGF2R* reduces epithelial barrier integrity and lysosomal acidification, *CXCL1*, *TNFα*, *PTGER4* and *USP27X* lowers inflammation and induces apoptosis. Immune responses in vivo: strong upregulation of *TLR1/2/3/6/7/9/13 Nod1*, *Nod2*, *Nlrp3*, *Nlrc4* and downstream NF-κB, MAPK, and Jak-STAT signaling, drives production of TNF-α, IL-1β, IL-6, IL-17, CXCL1, and CCL2, leading to extensive infiltration of neutrophils, macrophages, and dendritic cells. Solid and dotted arrows represent direct and indirect interactions, respectively. Results herein summarized relate to studies performed in lung cell infection models. Figure, not drawn to scale, was created with BioRender.com.

Once internalized within membrane-bound vacuoles, *A. baumannii* activates a series of finely tuned intracellular processes that determine its fate, ranging from cytotoxicity and autophagy control to the modulation of host transcriptional and signaling pathways to ensure its persistence within the host cells (Figure 3). The key role of OmpA in *A. baumannii* pathogenesis is underlined by its multiple and distinct functions during infection. Indeed, it not only anchors the *A. baumannii* to epithelial surfaces and participates to bacterial internalization, but also acts as a cytotoxic effector once internalized [62,80]. Following host cell uptake of OMVs mediated by the host GTPase dynamin-related protein 1 (DRP1), OmpA is translocated into mitochondria, where it disrupts membrane potential, triggers the release of cytochrome c and the production of reactive oxygen species (ROS), eventually leading to cell death [62]. In addition, OmpA fragments can reach the host cell nucleus by exploiting a nuclear localization signal, where they induce DNA fragmentation and chromatin condensation, ultimately promoting apoptosis and cell death [80].

### 3.4. The Autophagy Tug-of-War: Balancing Clearance and Persistence

Accumulating evidence suggests that OmpA-mediated signaling can also trigger autophagy via the mitogen activated protein kinases (MAPK)/c-Jun N-terminal kinase (JNK) pathways, thereby contributing to bacterial persistence within host cells and further subverting epithelial cell homeostasis [81]. However, the recent study by Woo et al. showed that OmpA can also inhibit autophagy of the host by blocking the phosphorylation of CaMKK2, and the following kinase cascade to ULK1 via AMPK, which is essential for autophagy induction [82]. These apparently divergent results may indicate a strain-dependent behavior (e.g., ATCC 19606 vs. ATCC 17978), the invasion time, or that OmpA can fine-tune multiple autophagic routes during infection [81,82]. Indeed, the targeted manipulation of the autophagic flux by OmpA represents a sophisticated and important signaling strategy to overcome cellular degradative defenses, thereby contributing to *A. baumannii* survival. Similarly to OmpA, Omp34 also contributes to cytotoxicity, as independently demonstrated by both Smani et al. [39] and Rumbo et al. [83]. These studies revealed that Omp34 triggers apoptosis via the intrinsic mitochondria-dependent pathway, involving the activation of caspase-9 and caspase-3, both in vivo and in human epithelial cells (HEp-2 and HeLa cell lines) [39,83]. Interestingly, Omp34 was shown to modulate host autophagy, as revealed by the accumulation of autophagy markers LC3-II and p62/SQSTM1, indicative of a blockade in autophagic flux in both cell lines; this impairment results in cytoplasmic vacuolization and increased cellular stress, ultimately contributing to cell death [83].

Following internalization into human lung epithelial cells, *A. baumannii* ATCC 17978 activates the host transcription factor EB (TFEB) to gain access to and persist within human lung epithelial cells; TFBE activation promotes bacterial survival by regulating host mechanisms like lysosomal biogenesis and autophagy, which bacteria exploit to avoid degradation and establish persistent infections [68]. In a following study, it was shown that the physiological expression of the autophagic protein synaptosome-associated protein 17 (STX17), a vesicular protein that regulates autophagolysosome formation, is disrupted upon *A. baumannii* invasion [84]. Under normal conditions, STX17 expression is positively controlled by the transcription factor Yin Yang 1 (YY1), together with TFEB, and negatively regulated by the long non-coding RNA growth arrest-specific transcript 5 (LncRNA-GAS5) [84]. During *A. baumannii* ATCC 19606 cell invasion, the bacterium inhibits the expression of YY1, leading to LncRNA-GAS5 overexpression, which in turn downregulates STX17 expression and further inhibits YY1 expression, eventually causing autophagy dysfunction and inflammation disorder [84]. Further evidence showed that invading *A. baumannii* ATCC 19606 can induce a complete, ubiquitin-mediated autophagic response both in epithelial cells and in vivo; this pathway was shown to be dependent on septins SEPT2 and SEPT9 and regulated through the Beclin-1/AMPK/ERK/mTOR signaling axis [85]. The bacterial enzyme isochorismatase BasF, involved in the production of the siderophore acinetobactin, was found to be critical for the recognition and clearance of intracellular *A. baumannii* ATCC 19606 and ATCC BAA1605 strains by autophagy, as its inactivation markedly reduced autophagic induction and bacterial clearance both in vitro and in vivo [85,86].

### 3.5. Rewiring the Host: Transcriptional and Metabolic Reprogramming

Another example of how *A. baumannii* hijacks host factors to promote infection is its ability to induce the transcription factor FOS, a key transcriptional regulator involved in cell proliferation, differentiation, inflammatory responses, and importantly, cell death (via apoptosis or necrosis), especially under stress or infection [87,88]. In particular, *A. baumannii* OMV components enhance the expression of the host enzyme tryptophan-2,3-dioxygenase (TDO) during infection, leading to increased production of kynurenine, an endogenous ligand for the aryl hydrocarbon receptor (AHR) that, upon activation, directly induces the transcription of the *FOS* gene; this interaction is the molecular basis for the cytotoxic responses in host epithelial cells [88]. Thus, it has been suggested that the impact on host tryptophan metabolism by *A. baumannii* causes AHR- and FOS-mediated cytotoxicity in infected cells, increasing host cell death [88].

Notably, the invasive ABC141 strain was recently shown to reside and replicate within mildly acidic, single-membrane vacuoles of human endothelial cells, without eliciting major signs of inflammation; these bacterial vacuoles were decorated with the typical late endosome or lysosomal marker LAMP1, despite lacking degradative lysosomal enzymes [89]. In addition, it was shown that ABC141 uses the type I secretion system (T1SS) to support intracellular multiplication and the T2SS to invade host cells, revealing for the first time the involvement of the T2SS in the intracellular life cycle of invasive *A. baumannii* [89]. During infection, host cells showed a hypoxic-associated response, but are unable to activate the classical Hypoxia-inducible factor 1 alpha (HIF1α) signaling pathway; instead, there is an upregulation of the lncRNA HIF1A-AS that represses HIF1α activation during hypoxic conditions [89]. Moreover, mitochondria accumulate close to the bacterial vacuoles, suggesting localized host metabolic remodeling that may favor bacterial persistence under nutrient-limiting and low-oxygen conditions [89].

The transcriptomic study provided by Crua Asensio et al. gives a temporal picture of human cervical epithelial cell responses to *A. baumannii* ATCC 15308 strain infection [90]. Initially, the few intracellular bacteria downregulate *COL5A1* and *IGF2R* genes, involved in collagen metabolism and intracellular trafficking, indicating an induced reduction in the cell monolayer barrier and low lysosome acidification [90]. As infection goes on, the impact of replicating bacteria on the host transcriptome becomes stronger, first upregulating genes involved in the immune and cytokine responses, followed by the downregulation of genes encoding pro-inflammatory mediators (*CXCL1*, *TNFα*, *PTGER4*) and anti-apoptotic molecules (*USP27X*), indicating bacterial modulation of host defenses to promote intracellular persistence [90]. Moreover, the upregulation of genes involved in oxidative stress and cytoskeletal remodeling suggests that host cells utilize ROS production and the stabilization of the cytoskeleton to overcome infection, to limit inflammatory damage, and to control bacterial internalization [90]. To deeply investigate how *A. baumannii* contrasts host immune defenses during infection, Zang et al. used a transcriptomic approach in a lethal murine pneumonia model in vivo model. [91]. Infection with the hypervirulent strain LAC-4 caused massive lung inflammation, tissue damage, and a high bacterial burden, associated with extensive infiltration of neutrophils, macrophages, and dendritic cells after 24 h, leading to severe tissue damage [91]. In accordance with the histopathology results, 3313 genes were upregulated; these include pattern recognition receptors, such as Toll-like receptors (*TLR*)*1/2/3/6/7/9/13*, as well as nucleotide-binding oligomerization domain (Nod)-like receptors (*NLRs*, *Nod1*, *Nod2*, *Nlrp3*, *Nlrc4*), indicating a broad activation of TLR–NLR signaling in response to infection. Also NF-κB, MAPK, and Janus kinase-signal transducer and activator of transcription (Jak-STAT) pathways were strongly activated, driving the production of cytokines and chemokines such as TNF-α, IL-1β, IL-6, IL-17, CXCL1, and CCL2. Additionally, the NLRP3 inflammasome and IL-1β signaling, and cell death pathways including apoptosis, pyroptosis, and necroptosis participate to the inflammatory response [91]. Remarkably, some microRNAs (e.g., *miR-155*, *miR-147*, *miR-6972*) were greatly upregulated, suggesting a post-transcriptional regulatory role in host inflammatory responses. On the other hand, 2975 genes were downregulated, including those belonging to the glycolysis/gluconeogenesis pathway, while oxidative phosphorylation and HIF-1 signaling were activated, indicating the increased requirement for energy and metabolic reprogramming of immune cells during infection [91].

Collectively, these findings indicate that the intracellular persistence of *A. baumannii* is not a uniform trait but a variable outcome influenced by genetic background, surface architecture, and host cell context. This variability underscores the ability of *A. baumannii* to establish complex intracellular interactions with epithelial and host immune cells to cause a deep remodeling of host pathways to pave the way for its adaptation to the respiratory microenvironment by inducing tissue inflammation, immune escape, and persistence within the lung niche. Altogether, the data reported reinforce the notion that this pathogen has evolved different adaptive strategies to persist within the host environment.

### 3.6. Hidden from the Hunter: Intracellular Persistence as a Barrier to Phage Therapy

Intracellular localization also represents a significant obstacle for bacteriophage therapy, as it physically shields bacteria from phage-mediated killing. Several studies have demonstrated the efficacy of bacteriophages and bacteriophage-antibiotic combinations against *A. baumannii* infections in animal pneumonia models [92,93]. Due to the low cellular uptake of phages, the ability of intracellular *A. baumannii* to reside within host cells further compromises phage efficacy in addition to other limitations (e.g., narrow host range, potential emergence of phage resistance, and inter-individual variability in host immune responses) [92,93]. To target these *A. baumannii* intracellular strains, the development of engineered phages equipped with cell-penetrating peptides, represents a promising strategy to target intracellular *A. baumannii* reservoirs [94].

## 4. Learning the Airway Rules: *A. baumannii* Adaptation

### 4.1. The Iron Will: Overcoming Nutritional Immunity

**Figure 4:**
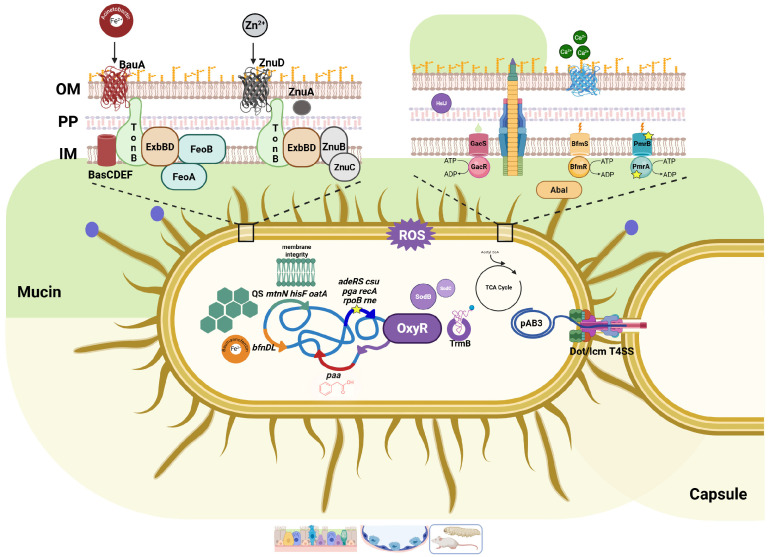
***A. baumannii* adaptation to the respiratory microenvironment.** The figure schematically illustrates how *A. baumannii* senses and adapts to the host respiratory niche. Nutrient acquisition: the acinetobactin system, including BasDF (biosynthesis) and BauA (transport), as well as *basBC*, sustains the infection and induces apoptosis. Additionally, the baumannoferrin A cluster, with *bfnDL*, works together with the Feo system, to grant survival in the host. The Znu system allows uptake of Zn^2+^. Environmental sensing and signaling: mucin acts both as a nutrient source and an extracellular signal, triggering the expression of virulence genes (*paa* operon, T6SS via GacS/GacA) and contributing to antibiotic tolerance. Against ROS, the bacterium activates mainly SodB over SodC, as well as the OxyR operon, including the periplasmic protein HslJ, and periplasmic chaperones. Moreover, for rapid post-transcriptional control, *A. baumannii* uses the TrmB methyltransferase. High Ca^2+^ concentrations upregulate expression of *ompA*, *bfmRS*, and *abaI* to increase virulence and bacterial invasion rate. Adaptation strategies: GacS/GacA regulation links central metabolism (TCA cycle), plasmid (pAB3) dissemination via Dot/Icm-like T4SS and virulence genes, including *paa* operon and T6SS. Other adaptation genes that confer protection against host factors include *mtnN*, *hisF*, *oatA*, quorum sensing components, and membrane integrity maintenance genes. Antibiotic and host pressure induce convergent mutations in global regulators (*pmrAB*, *adeRS*) and in virulence loci (*csu*, *pga*). These cause phenotypic changes (e.g., mucoid conversion, increased efflux pump expression, reduced outer membrane permeability), modifications of biosynthesis pathways for lipopolysaccharide and capsule, and upregulation of specific proteases, such as Clp, Lon (not represented). These changes enhance anti-phagocytic capacity, decrease antibiotic susceptibility, and promote persistence in the host. Results herein summarized relate to studies performed in bronchial and lung cell, *G. mellonella*, and murine infection models. Figure, not drawn to scale, was created with BioRender.com.

Beyond adhesion, invasion, and intracellular survival, *A. baumannii* also exhibits remarkable metabolic and regulatory adaptability that contributes to its adaptation to the harsh respiratory environment of the host (Figure 4). In addition to manipulating host metabolic pathways, *A. baumannii* survival and persistence depend on its ability to (patho)adapt to the intracellular niche; this adaptation involves extensive modifications of its metabolism and nutritional requirements, orchestrated by complex regulatory networks that include a plethora of global transcriptional regulators, several two-component systems, σ factors, and is further fine-tuned post-translationally by small RNAs [95]. A main challenge faced by intracellular *A. baumannii* cells is host metal sequestration (e.g., iron and zinc), a process known as nutritional immunity, combined with the high bacterial demand [8]. To overcome this constraint, *A. baumannii* senses metal limitation and expresses different metal homeostasis systems to capture these elements from metal-containing host proteins [96,97,98]. During epithelial infection, *A. baumannii* ATCC 19606 expresses both the biosynthetic BasD and transport BauA proteins of the acinetobactin-mediated iron acquisition system, required to sustain infection and induce epithelial cell apoptosis, highlighting its remarkable ability to adapt and persist in the host [79,95,99]. Furthermore, transcriptome analysis performed on bronchoalveolar lavages from in vivo infections with *A. baumannii* strains ATCC 17978 and the clinical isolate AbH12O-A2 revealed a strong upregulation of genes involved in iron uptake and host invasion [57]. As outlined before, *A. baumannii* strains defective in the *feoA* gene showed a significantly reduced ability to adhere to and invade host epithelial cells, underlining that the FeoA-mediated iron acquisition system contributes not only to bacterial adhesion but also to host cell invasion during pneumonia infection [57]. In strain ATCC 17978, genes associated with adhesion to A549 human alveolar epithelial cells and involved in iron uptake, including *feoA* (encoding the ferrous iron transport system component FeoA), *bnfL* (encoding an N-acetyltransferase, baumannoferrin biosynthetic protein), *basB* (encoding an acinetobactin biosynthesis protein), were also involved in in vivo virulence during pneumonia infection, along with *mtnN* (encoding an MTA/SAH nucleosidase), *yfgC* (renamed *bepA*, encoding a putative periplasmic metalloprotease), *hisF* (encoding the imidazole glycerol phosphate synthase cyclase subunit), and *oatA* (encoding an acyltransferase) [57]. Accordingly, recent transcriptomic and functional analyses performed in vivo in the *G. mellonella* infection model with the community strain Ab-C98 and the clinical reference strain ATCC BAA1605 showed a strong upregulation of genes belonging to the acinetobactin, baumannoferrin, and *feo* iron clusters [86]. Further functional validation by gene knockout showed that the deletion of the siderophore biosynthesis genes (*basC*, *bfnD*, and *basF*) significantly increased host survival by confirming that these determinants contribute to *A. baumannii* virulence. Notably, *basC* and *basF* are highly conserved among pathogenic *Acinetobacter* species, including *A. baumannii*, *A. pittii*, and *A. lactucae*, while *bfnD* is unique to *A. baumannii*, underlining the evolutionary specialization of this species for efficient iron acquisition and pathogenicity [86]. These data once again confirm the centrality of iron acquisition in *A. baumannii* pathogenesis. However, besides iron-uptake genes, the upregulation of genes such as *mtnN*, *bepA*, *hisF*, and *oatA* is highly suggestive of additional adaptation mechanisms, including the regulation of quorum sensing, maintenance of outer membrane integrity, modulation of host inflammatory responses, and protection against lysozyme-mediated degradation, further unveiling the multifaceted adaptive strategy of *A. baumannii* to persist within the respiratory microenvironment.

### 4.2. Reading the Mucus: Environmental Sensing and Genetic Plasticity

It has been reported that mucin, the heavily glycosylated and abundant protein secreted by goblet cells, exerts a dramatic impact on *A. baumannii* ATCC 19606T physiology within the airway environment, profoundly reprogramming its transcription profile [100]. For *A. baumannii*, mucin serves as both a nutrient source and an extracellular signal, which triggers the expression of virulence-associated genes, including the *paa* operon involved in the catabolism of phenylacetic acid, and the T6SS, through the GacS/GacA two-component system; indeed, it provides carbon, phosphate, and metal ions (Fe^2+^, Co^2+^, and Zn^2+^), reducing the bacterial need for siderophore production and metal efflux systems [100,101]. Hence, the mucin-rich milieu likely facilitates bacterial survival, promotes interactions with A549 human alveolar epithelial cells and contributes to antibiotic tolerance (e.g., reduced colistin efficacy), thereby allowing persistence and pathogenicity [100]. The selective pressure exerted by the host environment, iron restriction, and antibiotics rapidly reshapes *A. baumannii* genetic content and expression profile of key genes through different mechanisms, including point mutations, insertion sequence (IS) mobilization, deletions, duplications, and recombination events [102]. An elegant study by Wright et al. analyzed the genetic changes in genes occurring in 24 *A. baumannii* isolates retrieved from eight infected patients who received antibiotic treatment [102]. Convergent mutations among collected strains were found in the *pmrAB* and *adeRS* genes, most probably driven by antibiotic exposure (notably colistin and tigecycline), *csu* and *pga* biofilm-associated loci, as well as iron uptake systems; in addition, some isolates displayed broad transcriptomic shifts caused by a few mutations in global regulators (e.g., *recA*, *rpoB*, *rne*), underscoring how *A. baumannii* fine-tunes its gene expression to withstand antibiotics, acquire nutrients, and adapt to the host environment [102]. In addition, to face zinc limitation, *A. baumannii* possesses the Znu system, consisting of the inner membrane transporters, ZnuA, ZnuB, and ZnuC, as well as the outer membrane transporter, ZnuD, which mediates zinc uptake during in vivo lung infection [103]. Notably, the Znu system contributes to Zn^2+^ uptake even in the presence of calprotectin, a host-secreted zinc-binding protein released in response to bacterial infection [103].

### 4.3. Feeding the Invader: Central Metabolism and Carbon Sources

A nice demonstration of how *A. baumannii* can tightly coordinate its own central metabolism with virulence regulation upon sensing host environmental nutrient availability comes from how it modulates both plasmid dissemination and pathogenic potential [104]. Specifically, conjugation of the major plasmid pAB3 relies on a Dot/Icm-like type IV secretion system (T4SS), whose expression is co-regulated by the GacS/GacA two-component system with genes involved in central metabolic pathways, including those of the tricarboxylic acid (TCA) cycle. Therefore, the GacS/GacA system appears to be the most important signaling pathway directing the lifestyle shift in *A. baumannii*, indicating a strong link between metabolic sensing, horizontal gene exchange, and pathogenic fitness required for survival [104]. Notably, intracellular *A. baumannii* relies on host-derived, non-sugar carbon sources, mainly amino acids and organic acids, to sustain its metabolism within epithelial cells, a prerequisite for bacterial persistence [104]. Consistent with the asaccharolytic characteristics of the genus *Acinetobacter*, sugars are not preferentially used as carbon sources; instead, a broad spectrum of organic acids and amino acids is utilized [105,106]. Thus, these carbon sources directly enter the central metabolism via the TCA cycle, which acts as a central metabolic hub for energy production during *A. baumannii* intracellular survival [104]. Consequently, *A. baumannii* has the capacity to reprogram and optimize its metabolism depending on carbon sources to ensure intracellular survival and infection spread within the host [104].

### 4.4. Surviving the Storm: Oxidative Stress and Calcium Signaling

Other components of the airway microenvironment, such as calcium ions and oxidative stress, can also impact *A. baumannii* behavior. Notably, strains ATCC 19606 and *A. baumannii* AB5075 counteract oxidative stress by activating superoxide dismutases, thereby boosting bacterial fitness and extending survival during infection [100,107]. In particular, in strain AB5075, SodB was shown to play a predominant role over SodC in protecting bacteria from oxidative damage, supporting its persistence within epithelial cells [107]. In line with this, *A. baumannii* also relies on the enrichment of periplasmic chaperones, and redox-regulatory proteins that contribute to stress resistance and cellular homeostasis [38,108,109,110]. Furthermore, the tRNA methyltransferase TrmB was found to be essential to contrast the host oxidative environment in strains ARC6851 and Ab04 in a murine pneumonia model; these post-transcriptionally tRNA modifications, known as the tRNA “modificome,” add an additional and quick level of translation modulation that fine-tunes protein synthesis within the host airways [111]. Moreover, calcium concentration can influence host-*A. baumannii* interactions, in that high calcium concentrations were shown to increase the invasion rate of human respiratory epithelial cells by a clinical MDR *A. baumannii* strain [112]. Additionally, bacterial exposure to elevated calcium levels within the alveolar environment determined a significant upregulation of the *ompA*, *bfmRS*, and *abaI* genes, possibly indicating that calcium concentration promotes host–pathogen interactions [112]. These findings could justify the time-dependent cell death of human lung carcinoma cells by 113-16 and ATCC 19606 strains through the perturbation of host cytosolic calcium homeostasis, due to calpain and caspase-3 activation [113]. In addition, several adaptive mutations in key genes have been reported for *A. baumannii* ATCC 17978 during contact with human lung epithelial cells; these changes affect both morphology and phenotype, including mucoid conversion, increased ribosome translation, and efflux pump expression, as well as reduced outer membrane permeability [114]. Despite showing lower adhesion to lung epithelial cells, these genotype adjustments enhance bacterial antiphagocytic capacity, and significantly decrease antibiotic susceptibility, thereby promoting *A. baumannii* dissemination and persistence within the host [114]. Using an insertion sequencing (INSeq) approach, 157 genes were identified as being required to sustain the persistence of the ATCC 17978 strain in a mouse pneumonia model [115]. Among this pool, several genes encode well-known virulence factors, including those involved in lipopolysaccharide and capsule biosynthesis, amino acid and nucleotide metabolism, specific proteases such as Clp and Lon, and efflux pumps, as well as the integration host factor, a transmembrane lipoprotein, and proteins associated with the stress response [115]. On the other hand, only eight genes, encoding hypothetical proteins, were found to confer a disadvantage in this specific microenvironment, underlining the extensive genetic arsenal that *A. baumannii* employs to persist within the host [115]. Collectively, these findings highlight that iron acquisition and regulation represent a central hub in the *A. baumannii* adaptive strategy, linking metabolism, virulence, and persistence within the respiratory microenvironment.

### 4.5. A Tale of Two Cities: Niche-Specific Adaptations

While this review focuses on the respiratory epithelium, the urinary tract represents the second most frequent infection site for *A. baumannii* [24]. The urinary tract environment presents unique challenges, including high urea concentrations, low nutrient concentrations, extreme iron limitation, and intense shear stress generated by urine flow. Since adaptation is the most impressive weapon of *A. baumannii* pathogenesis, it exploits urea as a nitrogen source via urease activity to support growth; moreover, urea sensing promotes the upregulation of the acinetobactin-mediated iron acquisition system, centered on the BauA receptor, which is essential for systemic dissemination [24,116]. The metabolic adaptability of the uropathogenic *A. baumannii* UPAB1 strain is further complemented by site-specific adhesive strategies to resist mechanical clearance. In fact, adhesion is mediated by chaperone-usher pathway pili together with enhanced biofilm formation on both bladder surfaces and medical devices, such as catheters [24]. Although more studies are needed to fully understand these highly specialized adaptive mechanisms, they represent ideal targets for the development of effective therapeutic strategies against *A. baumannii* urinary tract infections [117].

## 5. Playing Hide-And-Seek: *A. baumannii* Immune Evasion Skills

### 5.1. Avoiding the First Line: Antimicrobial Peptides and Complement Evasion

As outlined before, *A. baumannii* can evade innate immune responses by modulating inflammatory signaling, and exploiting host cell death pathways [61,67,73,90,91,118,119]. The bacterial surface components, such as LPS, outer-membrane proteins, are actively recognized by pattern recognition receptors (PRRs) including TLR2, TLR4, and TLR9 that trigger the canonical NF-κB/MAPK signaling cascades to express cytokines (TNF-α, IL-6, IL-8) and chemokines (CXCL1, CXCL2, CCL2) that recruit neutrophils and monocytes to the airways [67,72,118,119,120]. Some *A. baumannii* strains can also trigger the NLRP3 inflammasome in macrophages and epithelial cells, resulting in the activation of caspase-1/11, release of IL-1β/IL-18 and pyroptosis, which induce further inflammation and programmed cell death; in parallel, invasive *A. baumannii* manipulates autophagic flux by delaying lysosomal fusion to allow intracellular persistence [91,121,122]. Recent evidence indicates that azithromycin, at high concentrations, is active against intracellular *A. baumannii* both in vitro and in vivo; this intracellular clearance activity seems to be linked to the induction of autophagosome formation and stimulation of host autophagy, thereby enhancing intracellular bacterial clearance [123]. Intriguingly, Nod1 was found not to be involved in the immune responses against *A. baumannii* infection in an in vivo model [124]. Conversely, Nod2 was shown to trigger the Nod2-serine/threonine-protein kinase 2 (Ripk2) pathways in lung epithelial cells, which activate downstream signaling, such as NF-κB and MAPKs, eventually promoting the production of cytokines or ROS to limit the intracellular growth of *A. baumannii* [125]. Additionally, Nod2-knockout mice showed elevated bacterial lung burdens at 4–12 h post infection, reduced ROS/reactive nitrogen species generation, and increased neutrophil influx, irrespective of strain virulence, indicating that Nod2 mediates early microbicidal oxidative responses [119]. Within host cells, the Nod2-signaling pathway is also responsible for the expression of β-defensins, peptides with potent anti-*A. baumannii* activity, thereby making Nod2 a central hub in antimicrobial peptide induction, oxidative burst, and balanced inflammation [125]. Besides β-defensins, 155 human anti-microbial peptides have been identified (https://aps.unmc.edu/home). Among these, cathelicidin LL-37, produced in airway epithelial cells, binds avidly to LPS and OmpA; through this interaction, LL-37 modulates LPS-mediated inflammation and inhibits ATP signaling–induced pyroptosis, thereby protecting experimental septic animals from death [126]. In addition, LL-37 plays an important role in limiting the bacterial burden by inducing the expression of cytokines and chemokines to attract neutrophils in response to *A. baumannii* infection [126,127,128,129]. The efficacy of these powerful molecules from human and non-human sources in the treatment of *A. baumannii* infections was thoroughly reviewed [130,131]. However, *A. baumannii* has evolved several strategies to resist the antibacterial activity of these peptides, including modifications of LPS lipid A, increased capsule expression, the maintaining of membrane integrity, as well as the reduction in permeability by decreasing OMP content [132]. Furthermore, the permanently active alternative pathway of the complement system represents an active defense against *A. baumannii* due to the spontaneous hydrolysis of the C3 component, enabling fast detection of the pathogen [133,134]. Factor H is a key regulator of this pathway, since it induces the decay of the alternative C3 convertase (C3bBb), thereby preventing the deposition of the opsonin C3b on host cell membranes; moreover, Factor H acts as a co-factor of Factor I, blocking the cascade by cleaving C3b and preventing the formation of new C3bBb complexes by competing with Factor B for binding to C3b [135,136]. Also in this case, *A. baumannii* has evolved strategies to evade complement-mediated killing, mainly by preventing or reducing C3b deposition on its surface via OmpA-Factor H binding and biofilm formation [15,32,137,138]. However, additional virulence factors have been implicated in complement evasion, including the OMP CipA, which interacts with plasmin to degrade fibrinogen and C3b, and the secreted serine protease PKF [11,134,138]. Beyond these surface-associated factors, several *A. baumannii* genes were found to be critical for the survival of *A. baumannii* in human serum, including *pntB*, *ddc*, *feoB*, and *fepA*, involved in peptidoglycan biosynthesis, redox and energy metabolism, and iron acquisition [139,140]. Also the *mla* genes, involved in the maintenance of the OM asymmetry in Gram-negative bacteria, were shown to be responsible for human complement-resistance; hence, the Mla-dependent OM integrity and stability seem to be at the basis of the protection from the lytic action of the host’s complement system [139]. Although further studies are needed to understand the strain-specific contribution of these complement interactors, it seems that these genes could cooperate to adopt the required metabolic and structural changes that enable the bacterium to overcome nutrient limitation, immune defenses, and host environmental stresses [133,140]. In addition, a marked difference was recently reported in how clinical *A. baumannii* isolates interact with the complement system [141]. It is known that all complement pathways converge on the generation of C5 convertase, which leads to the formation of the membrane attack complex (MAC) that can eventually lyse Gram-negative bacteria [142]. The study by Magda et al. reports that only strains carrying a specific capsule can block MAC deposition and resist serum killing, whereas others are vulnerable to complement-mediated lysis [141]. Furthermore, the highly variable exopolysaccharide capsule in *A. baumannii* causes a lower level of induction of pro-inflammatory cytokines, likely by limiting direct surface interactions and reducing phagocytosis, thereby impairing innate immune cell recruitment and bacterial clearance in vivo [143]. Indeed, capsulated *A. baumannii* strains elicit a weaker TLR4-dependent type I IFN response and evade phagocytosis by hindering effective interactions with immune cells [133]. In agreement with this, the *wza* mutant of strain Lac-4, lacking the polysaccharide export protein, was markedly more susceptible to killing by complement, whole blood, and human neutrophils than both the wild-type and a revertant mutant, underlining the critical role of the capsule in *A. baumannii* immune evasion and pathogenicity [144].

### 5.2. Turning the Tables: Neutrophils, Macrophages, and Phagocytic Escape

A cornerstone in *A. baumannii* defense at the early stage of infection is the local recruitment of neutrophils [6,119,133,145]. Indeed, neutrophils can respond to *A. baumannii* infections through phagocytosis, degranulation, and the oxidative burst [119,134,146]. It has been reported that human neutrophils can kill *A. baumannii* within 4 h after infection; genetic expression studies highlighted that neutrophil-*A. baumannii* interactions activate the TLR4-NF-kB pathway to secrete pro-inflammatory mediators to attract additional immune cells to the site of infection [147]. Moreover, the neutrophil oxidative burst mainly relies on the nicotinamide adenine dinucleotide phosphate oxidase (NADPH); this enzyme influxes superoxide into the phagosomal membrane, which is then converted into hydrogen peroxide, and then converted by the myeloperoxidase into hypochlorous acid, detrimental for *A. baumannii* [134,146]. Neutrophils possess an additional killing mechanism against pathogenic bacteria through the release of neutrophil extracellular traps (NETs) or NETosis, which occurs when neutrophils die, leading to the formation of sticky, net-like structures composed of unfolded chromatin and lysosomal enzymes that capture and kill bacteria [133,148,149]. However, *A. baumannii* ATCC 19606 was able to inhibit the formation of NETs by downregulating the expression of the CD11a surface receptor, which is essential for the adhesion step required to initiate NETosis, thereby prolonging neutrophil survival [150]. Furthermore, human neutrophils can produce peptides or α-defensins as an extra strategy for non-oxidative killing of phagocytosed bacteria [151]. Recently, Liao et al. reported high concentrations of human neutrophil peptides (HNPs) 1-3, differing by only one amino acid residue at the N-terminus, in bronchoalveolar lavage fluids or sputum from patients with *A. baumannii* pneumonia [152]. Surprisingly, HNP1 promotes *A. baumannii* ATCC 19606 adhesion to human bronchial, lung, and larynx cell monolayers within minutes in a dose-dependent manner, acting directly via the OmpA protein [152]. Hence, this HNP1-OmpA interaction enhances biofilm formation, allowing *A. baumannii* to become more tolerant to antibiotics and more capable of colonizing host tissues; noteworthy, this effect does not directly correlate with an increased expression of biofilm-related genes, but instead it seems to indirectly modulate metabolic pathways that favor persistence and increase antibiotic-resistance [152]. These unexpected findings contrast with the well-known protective role of HNP1 in innate immunity, further underlining how *A. baumannii* has evolved a strategy to subvert host defense and promote its persistence.

Conversely, the contribution of tissue-resident macrophages in the host-*A. baumannii* defense is more complex and variable than that of neutrophils [134,146,149,153]. Indeed, macrophages are among the first innate cells present at the infection site before large numbers of neutrophils are recruited [134,146,149]. Upon encountering bacteria, macrophages rapidly activate via the TLR4-dependent response [154]. Although the role of TLR2 in recognizing *A. baumannii* is still not well elucidated, some evidence showed that macrophage activation can also be mediated by the TLR2/MyD88/NF-κB pathway [155]. In both cases, this activation leads to the M1 phenotype, characterized by the strong production of pro-inflammatory cytokines and chemokines, including macrophage inflammatory protein 2 (MIP-2), IL-6, and TNF-α, essential for the recruitment of neutrophils; in addition, activated macrophages produce ROS in response to *A. baumannii* infection as a bactericidal mechanism [134,146,149]. Also, a moderate expression of the inducible nitric oxide synthase was reported in response to *A. baumannii* infection that leads to the production of nitric oxide, which is endowed with a powerful antimicrobial activity [146]. *A. baumannii* phagocytosis by macrophages is very fast, within minutes in vitro and within a few hours in vivo, thereby contributing to the early containment of bacterial spread [109,156]. Nonetheless, despite this early involvement, several studies suggest that alveolar macrophages play a limited role in the ultimate clearance of the pathogen, while others indicate that macrophages are important early responders during *A. baumannii* infection, as their depletion markedly increases susceptibility to respiratory infections [134,146,149]. The study by Sycz et al. demonstrated that the clinical urinary isolate UPAB1 can persist and replicate within macrophage vacuoles during the early phase of infection, eventually inducing macrophage lysis and being released into the extracellular environment [157]. This intracellular behavior occurs through T1SS-dependent effector secretion and the expression of multiple chromosomally encoded virulence factors regulated by the large conjugative plasmid pAB5 [157]. Furthermore, it was shown that primary macrophages were able to efficiently kill both ATCC 19606T and ACICU strains without eliciting a strong inflammatory response; however, a small subset of them infected with ACICU allowed intracellular vesicular replication, eventually leading to macrophage lysis [158]. Thus, the debated role of the bactericidal activity of macrophages could be explained by considering strain-specific differences, with clinical or epidemic isolates displaying greater persistence and an enhanced ability to trigger inflammatory signaling, as in the case of ACICU, which induced significantly lower levels of the anti-inflammatory cytokine IL-10 than ATCC 19606T [158]. Indeed, IL-10 levels control macrophage phagocytosis and bactericidal activity via JAK-STAT3 signaling, as shown in mice infected with *A. baumannii* [153]. The JAK-STAT3 pathway is also required for the optimal expression of CD80 and CD86, which enhance antigen presentation and regulate the subsequent activation and differentiation of adaptive immune responses [159]. Therefore, the ability of some *A. baumannii* isolates, such as ACICU, to manipulate the macrophage response underscores how *A. baumannii* has developed several effective strategies to evade innate immunity defenses, despite the robust antimicrobial functions of neutrophils and macrophages [158]. Another major mechanism contributing to *A. baumannii* host persistence is its resistance to ROS, through the elevated activity of enzymes under the control of the transcriptional regulator OxyR, which markedly limits oxidative killing [108,109,110]. In addition, besides being the mechanism for surviving under manganese starvation, the transcriptional regulator MumR also contributes to *A. baumannii* protection against ROS [160]. Indeed, MumR-regulated gene products are involved in phenylacetic acid metabolism, whose intermediates can attract neutrophils to the site of infection; the combined activity of these enzymes and the *paa* operon can lower phenylacetate levels, thereby delaying and limiting neutrophil migration to the site of infection [146,160]. Furthermore, *A. baumannii* can escape from phagosomes or directly induce host cell lysis by damaging their membranes via the action of phospholipases C and D [161]. However, other mechanisms can assist *A. baumannii* colonization. For instance, it was reported that the clinical isolate Ci79 can exploit the mucosal immunoglobulin A (SlgA) to enhance its epithelial colonization via the bacterial protein thioredoxin A (TrxA), which reduces disulfide bonds in SIgA and compromises its barrier function [162]. While this was demonstrated in the gastrointestinal tract, such a mechanism could plausibly occur in airway environments as well. Overall, these findings collectively demonstrate that *A. baumannii* has a remarkably complex arsenal of immune evasion strategies targeting virtually every layer of the respiratory innate defense, from reshaping inflammatory responses and inflammasome activation to autophagy, complement, neutrophil function, and macrophage-mediated clearance, being able to resist oxidative and non-oxidative killing, hijacking host cell death pathways, and taking advantage of host molecules. This broad adaptability further stresses the urgent need for effective therapeutic approaches capable of counteracting these sophisticated evasion mechanisms.

### 5.3. Beyond the Petri Dish: Limits of Current In Vitro Models

It is important to highlight that much of the current data on *A. baumannii* derives from in vitro studies using immortalized or cancerous epithelial cell lines (mainly the A549 cell line). While these models have been extremely useful in the past for understanding the molecular mechanisms of several pathogens, they fail to recapitulate the architecture, cellular diversity, or microenvironment of the human airway; in fact, cell lines exhibit uncontrolled proliferative growth and a dedifferentiated phenotype, which may limit the physiological relevance of the findings [163]. Currently, several physiologically relevant models have been developed, including primary human airway epithelial cells cultured at the air–liquid interface, organoid systems, and lung-on-a-chip platforms, which can also be integrated with immune cell co-cultures to reach an even higher degree of physiological complexity [61,163]. The broader use of such advanced platforms that better mimic the cellular complexity, polarization, and mucus production of the respiratory epithelium could significantly boost our knowledge on *A. baumannii* pathogenesis and accelerate the development of effective therapeutic interventions.

## 6. Conclusions and Future Directions

Adhesion, invasion, and intracellular persistence of *A. baumannii* within host cells, mainly the respiratory epithelium, represent important survival strategies that substantially contribute to its success as a nosocomial pathogen. The associated multidrug-resistance phenotype has made this opportunistic pathogen a worldwide concern for almost the last 10 years [4]. The data reported in this review highlight that the interaction of *A. baumannii* with host epithelia is not a static event but rather a dynamic process that forces the bacterium to undergo genetic and metabolic adaptations in order to survive and persist in the host. Despite extensive research efforts, several steps in *A. baumannii* pathogenesis, from adhesion to intracellular trafficking and long-term intracellular survival, remain largely uncharacterized. Moreover, it is increasingly evident that there is a dramatic heterogeneity in the behavior and pathogenicity between *A. baumannii* lab-domesticated strains vs. clinical isolates. Since the pathogenic strategies and persistence capabilities are intrinsically linked to their genetic diversity and their specific interactions with host epithelia, there is an urgent need for scientific consistency and standardization in research efforts. Thus, achieving common and shareable results will require a coordinated global commitment to share well-characterized clinical strains, harmonize infection and analysis protocols, and adopt common, advanced experimental models. We strongly believe that inter-laboratory variability can be overcome through a collaborative approach aimed at accurately mapping the relationship between bacterial genotype and the host-interaction phenotype. Integrating these features with Artificial Intelligence could represent a new frontier for uncovering common traits and/or molecular signatures correlated with specific patterns of *A. baumannii* host pathogenesis. Potentially, this could predict the strain-specific severity of the infection, guiding clinical options. In addition, elucidating the key players of *A. baumannii* adhesion, invasion, resistance to phagocytosis, and evasion of phagocytic killing will boost studies aimed at blocking these processes through the development of innovative anti-virulence therapies that, instead of killing the bacteria, disarm their pathogenic features. These kind of strategies are endowed with the tremendous advantage of exerting less selective pressure, thereby significantly reducing the risk of generating new resistant strains, unlocking next-generation therapeutics.

## Figures and Tables

**Table 1:** Summary of known *A. baumannii* adhesins and invasins, their role and pathogenic outcome, and their host targets.

Bacterial Factor	Strain	Experimental Model	Role and Pathogenic Outcome	Host Target	Reference
sRNA 13573	ATCC 17978	In vitro (A549 cells)	Regulates *csuE* expression; modulates pili production and biofilm formation	Unknown	[14]
LH92_11085	MAR002	In vitro (A549 cells)	FimA-like subunit; forms long pili that promote adhesion to alveolar cells	Unknown	[16]
PdeB	ATCC 17978, AB5075	In vitro (A549 cells); In vivo (BALB/c mice)	Phosphodiesterase that enhances CsuA/B pili expression, promoting cell adhesion	Unknown	[17]
ComC	AB AYE-T	In vitro (HUVEC cells)	Acts as a T4aP tip adhesin to promote bacterial attachment to host cells.	Unknown	[18]
PilA	*A. nosocomialis* M2	In vitro (A549 cells)	Major pilin T4aP; promotes cell adhesion	Unknown	[19]
Ata	Multiple strains	In vitro (HUVEC cells, binding assays with beads); In vivo (*G. mellonella*, C57BL/6 mice)	Trimeric autotransporter; mediates binding to ECM components	Collagen, Laminin, Fibronectin	[20,21,71]
InvL	UPAB1	In vitro (MDCK and 5637 cells) In vivo (C57BL/6 mice)	Member of T2SS; essential for cell entry and surface colonization	α5β1 integrin, Fibronectin	[22]
AbFhaB/FhaC	AbH12O-A2	In vitro (A549 cells); In vivo (*Caenorhabditis. elegans* and BALB/c mice)	Filamentous hemagglutinin proteins involved in ECM binding and cell adhesion.	Fibronectin	[25]
OmpA	ATCC 19606, ATCC 17978	In vitro (HEp-2, HeLa and A549 cells); In vivo (C57BL/6 mice)	Major porin; triggers zipper-like invasion and host cell apoptosis	Fibronectin, TLR2	[31,34]
Omp34	ATCC 17978, ATCC 19606, strain 58ST	In vitro (A549, RAW246.7 and HeLa cells) In vivo (C57BL/6 and BALB/c mice)	Mediates attachment and internalization; induces vacuolization	Fibronectin	[39,41]
TonB-dependent copper receptor	ATCC 19606	In vitro (A549 cells); In vivo (BALB/c mice)	Promotes adhesion by binding to ECM components	Fibronectin	[34,40]
OmpW	ATCC 17978	In vitro (A549 cells); In vivo (C57BL/6 mice)	Promotes both cell adhesion and invasion	Unknown	[44]
CarO	ATCC 19606, AB5075, ATCC 17978	In vitro (A549, WI38 and HNEpC cells); In vivo (BALB/c and C57BL/6 mice)	Promotes both cell adhesion and invasion	Unknown	[35,47,48]
OccAB1 (OprD, Chop)	ATCC 17978, AB5075	In vitro (A549 cells) In vivo (C57BL/6 mice)	Porin modified by ChoP; exploits host signaling for cellular entry	PAFR	[35,51]
YiaD	AB5075	In vitro (A549 cells)	Promotes cell adhesion	Unknown	[35]
BamA	ATCC 19606, strain 58ST	In vitro (A549 cells); In vivo (BALB/c mice)	Component of β-barrel assembly; promotes microfilament-dependent invasion	Cytoskeleton components	[55]
OmpH	*A. baumannii* genome collection	In silico model	Bioinformatically inferred adhesin	Unknown	[56]
FimF	*A. baumannii* genome collection	In silico model	Bioinformatically inferred adhesin	Unknown	[56]
FeoA	ATCC 17978, AbH12O-A2	In vitro (A549 cells); In vivo (BALB/c mice)	Ferrous iron transport system; essential for adhesion and pneumonia onset	Unknown	[57]
Hcp/VgrG	ATCC 17978	In vitro (HPAEpiC cells)	Core components of the T6SS; contribute to initial attachment to host cells	Unknown	[58]
Wza	ATCC 17978, strain SKLX024256	In vitro (A549 cells) In vivo (*G. mellonella* and BAMH/C mice)	Capsule export protein; required for environmental persistence and adhesion	Unknown	[59]
TatABC	ABC141	In vitro (A549 and EA.hy 926 cells)	Twin-arginine translocation (Tat) export system; promotes cell adhesion	Unknown	[60]

## Data Availability

Data sharing not applicable to this article as no datasets were generated or analyzed.
